# Ferroptosis and its role in osteoarthritis: mechanisms, biomarkers, and therapeutic perspectives

**DOI:** 10.3389/fcell.2024.1510390

**Published:** 2024-12-18

**Authors:** Shanyu Lu, Zhenyu Liu, Meiling Qi, Yingchao Wang, Le Chang, Xiaolong Bai, Yingguang Jiao, Xinyao Chen, Junping Zhen

**Affiliations:** ^1^ College of Medical Imaging, Shanxi Medical University, Taiyuan, Shanxi, China; ^2^ Department of Imaging, Second Hospital of Shanxi Medical University, Taiyuan, Shanxi, China; ^3^ Shanxi Key Laboratory for Immunomicroecology, Taiyuan, Shanxi, China; ^4^ Molecular Imaging Laboratory, Second Hospital of Shanxi Medical University, Taiyuan, Shanxi, China

**Keywords:** osteoarthritis, ferroptosis, molecular mechanisms, biomarkers, therapeutic strategies

## Abstract

Osteoarthritis (OA) is one of the leading causes of disability worldwide, characterized by a complex pathological process involving cartilage degradation, synovial inflammation, and subchondral bone remodeling. In recent years, ferroptosis, a form of programmed cell death driven by iron-dependent lipid peroxidation, has been recognized as playing a critical role in the onset and progression of OA. Investigating the molecular mechanisms of ferroptosis and its involvement in OA may offer novel strategies for diagnosing and treating this disease. This review first outlines the core mechanisms of ferroptosis, with a particular focus on the roles of critical molecules such as Glutathione Peroxidase 4 (GPX4), Transferrin Receptor 1 (TfR1), and Nuclear Receptor Coactivator 4 (NCOA4). Subsequently, this study examines the specific impacts of ferroptosis on the pathophysiology of OA. Building on this, the potential of ferroptosis-related biomarkers for OA diagnosis and treatment is highlighted, along with proposed therapeutic strategies targeting ferroptosis regulation. This review aims to deepen the understanding of ferroptosis mechanisms and advance the clinical application of regulatory therapies for OA.

## 1 Introduction

Osteoarthritis (OA) is a highly prevalent and destructive degenerative joint disease, affecting approximately 7.6% of the global population, with over 595 million cases in 2020 alone (GBD, 2021 Osteoarthritis Collaborators, 2023). Its incidence is expected to approach 1 billion by 2050, driven by the aging population (GBD, 2021 Osteoarthritis Collaborators, 2023). This rising prevalence poses a significant public health challenge (Martel-Pelletier et al., 2016; Grandi and Bhutani, 2020). The pathological features of OA include cartilage degeneration, synovial inflammation, subchondral bone remodeling, and joint space narrowing, among others (Barnett, 2018; Zhou et al., 2024). Traditionally, OA has been attributed primarily to excessive mechanical load; however, recent research indicates that it is a multifactorial process involving inflammation, metabolic imbalance, and impacts on various joint structures (Barnett, 2018; Hunter and Bierma-Zeinstra, 2019; Zhou et al., 2024). Despite extensive studies revealing some molecular mechanisms and risk factors associated with OA, the exact pathogenesis remains unclear, and effective treatments to halt or slow disease progression are lacking (Henrotin, 2022; Salman et al., 2023). Clinically, OA management predominantly focuses on symptom relief and surgical intervention, underscoring the urgent need to explore its underlying mechanisms and identify new therapeutic targets (Hunter et al., 2020; Kolasinski et al., 2020; Cho et al., 2021; Loughlin, 2022; Mobasheri et al., 2023).

With advancements in the study of cell death mechanisms, ferroptosis—a form of cell death distinctly different from traditional apoptosis and necrosis—has been identified (Dixon and Pratt, 2023; Wahida and Conrad, 2023; Berndt et al., 2024). Ferroptosis involves iron-dependent lipid peroxidation, leading to disrupted iron homeostasis, excess ROS production, and increased lipid peroxidation byproducts (Chen et al., 2023; Deng et al., 2023; von Krusenstiern et al., 2023; Bell et al., 2024; Dixon and Olzmann, 2024). Ferroptosis, discovered in 2012, is now recognized as a key factor in various diseases, including neurodegenerative disorders, cancer, and ischemia-reperfusion injury (Dixon et al., 2012; Deng et al., 2023; Fang et al., 2023; Graham et al., 2023; Miao et al., 2023; Yao et al., 2023; Ahola and Langer, 2024; Levi et al., 2024). Initial research indicates that ferroptosis could be a primary mechanism responsible for OA’s death of chondrocytes and synoviocytes (Miao et al., 2022; He Q et al., 2023). Increased intra-articular iron levels, lipid peroxidation, and weakened antioxidant defenses promote ferroptosis, worsening cartilage degeneration, and joint function loss (Chen J et al., 2024). This finding provides fresh insight into the pathogenesis of OA and presents potential for developing new therapeutic approaches aimed at targeting ferroptosis.

Although research on the precise mechanisms and clinical relevance of ferroptosis in OA is still in its infancy, the volume of related studies is growing swiftly (Yang et al., 2021; Ohnishi et al., 2022; Wang et al., 2023). A thorough review of the molecular pathological mechanisms, potential physiological functions, and therapeutic possibilities of ferroptosis in OA is thus essential. This review systematically outlines the core mechanisms of ferroptosis and its specific impacts on OA, explores the potential of ferroptosis-related biomarkers as diagnostic and therapeutic targets, and discusses the challenges and opportunities for clinical translation.

## 2 Biological mechanisms of ferroptosis

### 2.1 Overview of ferroptosis

Ferroptosis is a distinct type of programmed cell death, setting itself apart from conventional forms like apoptosis, necrosis, and autophagy. Its uniqueness lies in its dependence on iron accumulation, which triggers lipid peroxidation (Dixon et al., 2012; Chen et al., 2016; Zhang et al., 2018; Frank and Vince, 2019; Stockwell and Jiang, 2020; Liu et al., 2022; Tang et al., 2024). Morphologically, ferroptosis is characterized by alterations in mitochondrial structure, such as increased mitochondrial membrane density, diminished or missing cristae, and overall mitochondrial shrinkage (Wang et al., 2017; Fang et al., 2019; Deng et al., 2023; Mao et al., 2023). Biochemically, ferroptosis is indicated by the inhibition of cystine/glutamate antiporter (System Xc^−^), the depletion of glutathione (GSH), decreased activity of glutathione peroxidase 4 (GPX4), accumulation of lipid peroxides (LOOH), and a decline in mitochondrial membrane potential (Mao et al., 2021; Wang and Min, 2021). These changes result in excessive intracellular iron accumulation, triggering oxidative stress and cellular damage (Ingold et al., 2018; Bersuker et al., 2019; Zheng and Conrad, 2020).

Current research on the mechanisms of ferroptosis focuses on three primary signaling axes: dysregulated iron metabolism, lipid peroxidation, and the System Xc^−^-GSH-GPX4 axis. Dysfunctions in these pathways can lead to cellular iron overload. To maintain iron homeostasis, cells employ intricate regulatory mechanisms, including iron uptake via the transferrin receptor (TFR1), storage of iron in ferritin, and ferritinophagy to mitigate the toxic effects of iron overload (Kakhlon and Cabantchik, 2002; Gammella et al., 2015; Anderson and Frazer, 2017; Feng et al., 2020). GPX4 plays a critical role in counteracting ferroptosis by mitigating lipid peroxidation, while other factors like nicotinamide adenine dinucleotide phosphate (NADPH) and coenzyme Q10 (CoQ10) also contribute to ferroptosis regulation (Bersuker et al., 2019).

**FIGURE 1:**
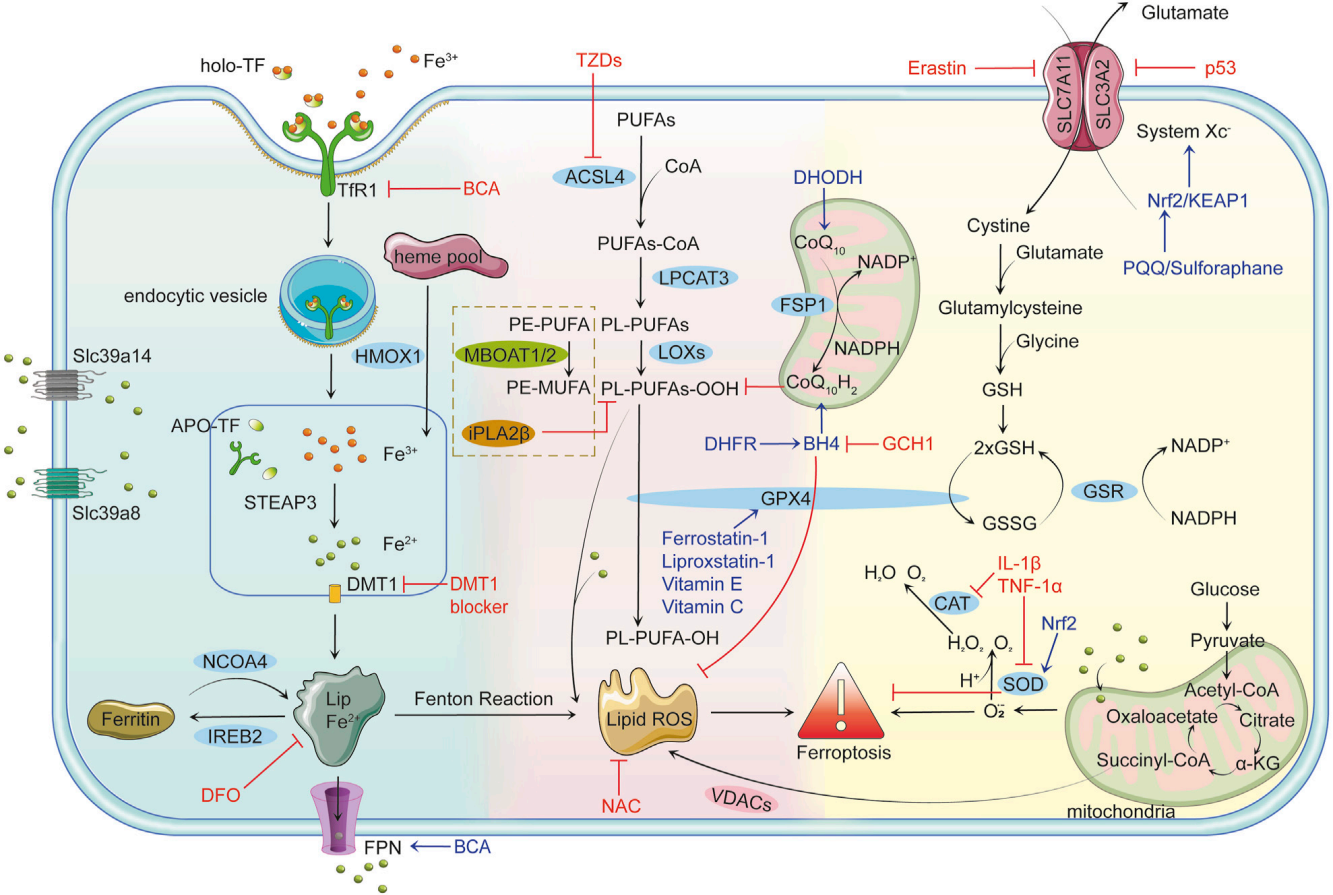
Mechanisms of Ferroptosis Iron Metabolism and Ferroptosis: Cells uptake iron-bound transferrin (TF) through transferrin receptor 1 (TFR1). Iron enters the cell in the Fe³⁺ form via endocytosis and is reduced to Fe^2^⁺ by six-transmembrane epithelial antigen of prostate 3 (STEAP3) in the endosome, subsequently transported to the cytoplasm by divalent metal transporter 1 (DMT1). Non-transferrin-bound iron (NTBI) uptake, mediated by solute carrier family 39 member 14 (Slc39a14) and solute carrier family 39 member 8 (Slc39a8), further contributes to intracellular iron overload. Free Fe^2^⁺ can participate in the Fenton reaction, producing highly reactive hydroxyl radicals (•OH) that trigger lipid peroxidation and lead to the accumulation of lipid reactive oxygen species (Lipid ROS). Ferritin alleviates iron toxicity by storing iron, while Nuclear Receptor Coactivator 4 (NCOA4) mediates ferritin degradation, releasing iron ions and enhancing ferroptosis. Lipid Peroxidation and Ferroptosis: Polyunsaturated fatty acids (PUFAs) are catalyzed by long-chain fatty acid-CoA ligase 4 (ACSL4) to form PUFAs-CoA, which are then integrated into the lipid bilayer by lysophosphatidylcholine acyltransferase 3 (LPCAT3), producing phospholipid PUFAs (PL-PUFAs). Lipoxygenases (LOXs) further oxidize PL-PUFAs into phospholipid peroxides (PL-PUFA-OOH), and the accumulated lipid ROS triggers ferroptosis. Voltage-dependent anion channels (VDACs) facilitate the accumulation of lipid ROS, accelerating cell death. Glutathione peroxidase 4 (GPX4)-independent pathways, such as iPLA2β (a calcium-independent phospholipase A2 family member) and membrane-bound O-acyltransferase 1/2 (MBOAT1/2), provide additional defense against ferroptosis by reducing peroxidized lipids and remodeling phospholipids, respectively. Antioxidant Defense Mechanisms and Ferroptosis: GPX4 is the primary inhibitor of ferroptosis, reducing PL-PUFA-OOH to non-toxic phospholipids (PL-PUFA-OH) using reduced glutathione (GSH) to prevent lipid ROS accumulation. GSH synthesis depends on System Xc^−^, which consists of solute carrier family seven member 11 (SLC7A11) and solute carrier family three member 2 (SLC3A2) and facilitates the transmembrane exchange of cystine and glutamate. Inhibition of System Xc^−^ decreases GSH production, leading to lipid peroxidation and ferroptosis. Key Regulatory Factors and Drug Actions: p53 limits GSH synthesis by inhibiting System Xc^−^, promoting ferroptosis. The nuclear factor erythroid 2-related factor 2 (Nrf2)/kelch-like ech-associated protein 1 (KEAP1) pathway enhances cellular antioxidant capacity and inhibits ferroptosis. Tetrahydrobiopterin (BH4), synthesized by gtp cyclohydrolase 1 (GCH1), boosts antioxidant defenses and suppresses lipid peroxidation. Ferroptosis suppressor protein 1 (FSP1) participates in antioxidant reactions through coenzyme Q10 (CoQ10), inhibiting ferroptosis. Erastin promotes ferroptosis by suppressing System Xc^−^ and reducing GSH levels. Ferrostatin-1 and Liproxstatin-1 prevent ferroptosis by inhibiting lipid peroxidation, while deferoxamine (DFO) chelates free iron to prevent ferroptosis by blocking the Fenton reaction. N-acetylcysteine (NAC) enhances antioxidant capacity by increasing GSH synthesis. Thiazolidinediones (TZDs) reduce lipid peroxide formation by inhibiting ACSL4 activity. Connection between Ferroptosis and Inflammatory Responses: Pro-inflammatory factors such as interleukin-1β (IL-1β) and tumor necrosis factor-alpha (TNF-α) can induce ROS production, facilitating ferroptosis. Concurrently, ferroptosis can activate inflammatory responses, exacerbating cellular damage. Role of Mitochondria in Ferroptosis: Mitochondria produce energy via oxidative phosphorylation and the tricarboxylic acid (TCA) cycle while generating superoxide (O₂•⁻), which is converted to H₂O₂ by superoxide dismutase (SOD). H₂O₂ reacts with Fe^2^⁺ to form OH•, further aggravating lipid peroxidation. Additionally, mitochondria release ROS through VDACs, closely linking this process to ferroptosis. Dihydroorotate dehydrogenase (DHODH) regulates ferroptosis by influencing mitochondrial redox reactions.

In addition to these well-characterized mechanisms, recent studies have highlighted alternative ferroptosis surveillance pathways that operate independently of GPX4. Enzymes such as FSP1 and MBOAT1/2 have been identified as important contributors to these pathways, suggesting a broader and more complex regulatory network of ferroptosis beyond the conventional System Xc^−^-GSH-GPX4 axis (Liang et al., 2023). The complex biological network governing ferroptosis highlights its central role in cellular homeostasis and disease progression. This understanding provides a crucial foundation for exploring ferroptosis-driven mechanisms in osteoarthritis. Figure 1 offers a comprehensive depiction of the critical molecular mechanisms underpinning ferroptosis.

#### 2.1.1 Iron metabolism

Iron metabolism and regulation are crucial for maintaining normal cellular functions (Ward et al., 2014; Zheng and Conrad, 2020; Simao and Cancela, 2021). Transferrin receptor 1 (TfR1) mediates the endocytosis of iron-bound transferrin (TF) (Aisen et al., 1978; Johnsen et al., 2019). TF, a natural chelator abundant in plasma, possesses two high-affinity iron-binding sites. Once TF binds to Fe³⁺ to form holo-TF, it associates with TfR1 on the cell surface and is internalized. In the acidic environment of endosomes, the lowered pH facilitates the release of Fe³⁺ from the Tf-TfR1 complex, allowing TfR1 and apotransferrin (apoTF) to be recycled back to the cell surface (Klausner et al., 1983; Zhou et al., 2021). During this process, divalent metal transporter 1 (DMT1) is crucial for the transmembrane transport of iron, primarily responsible for transporting iron ions from the extracellular space or organelles into the cytoplasm. Metalloreductase six-transmembrane epithelial antigen of prostate 3 (STEAP3) possesses ferrireductase activity, reducing Fe³⁺ to Fe^2^⁺, which is then released into the cytoplasm via DMT1, contributing to an unstable iron pool (Pantopoulos, 2004; Feng et al., 2020; Koike et al., 2020). Excess iron is stored in ferritin or exported extracellularly through ferroportin 1 (FPN1) (Kakhlon and Cabantchik, 2002).

Intracellular iron overload is a key driver of ferroptosis, facilitated by increased iron uptake and ferritin degradation through ferritinophagy. In ferroptosis-sensitive cells, TfR1 expression is upregulated, enhancing iron uptake, while ferritin heavy chain (FTH1) and ferritin light chain (FTL) undergo selective degradation via ferritinophagy (Santana-Codina and Mancias, 2018; Li et al., 2024). This process is regulated by nuclear receptor coactivator 4 (NCOA4), a critical mediator of intracellular iron homeostasis. NCOA4 recognizes and binds to ferritin, transporting it to lysosomes for degradation (Latunde-Dada, 2017). This releases stored iron, increasing the pool of bioavailable iron (Mancias et al., 2015). Upregulation of NCOA4 significantly elevates intracellular iron levels, further promoting lipid peroxidation and ferroptosis (Masaldan et al., 2018). Additionally, research has found a close interplay between NCOA4 and other ferroptosis-related signaling pathways. For instance, NCOA4-mediated ferritinophagy may impact the activity of glutathione peroxidase 4 (GPX4), as increased iron levels can deplete intracellular antioxidants like glutathione, indirectly inhibiting GPX4 function and thereby facilitating ferroptosis (Mancias et al., 2014).

Additionally, iron overload in ferroptosis is further modulated by key metal ion transporters, including solute carrier family 39 member 14 (Slc39a14) and solute carrier family 39 member 8 (Slc39a8) (Aierken et al., 2024). Slc39a14 primarily facilitates the uptake of non-transferrin-bound iron (NTBI), significantly contributing to intracellular iron accumulation (Aierken et al., 2024). Likewise, Slc39a8 also has a similar function, enhancing iron import and increasing intracellular iron levels (Aierken et al., 2024). Upregulation of these transporters promotes iron overload, lipid peroxidation, and ferroptosis. Conversely, inhibiting Slc39a14 or Slc39a8 has alleviated ferroptosis by reducing intracellular iron levels and mitigating oxidative stress (Aierken et al., 2024).

Together, the processes of ferritinophagy, mediated by NCOA4, and enhanced iron import via Slc39a14 and Slc39a8 synergistically increase intracellular iron levels, solidifying their roles as critical factors in regulating ferroptosis.

#### 2.1.2 Lipid peroxidation

Lipid peroxidation, a central driving process of ferroptosis, is a key distinguishing feature from other forms of cell death (Liang et al., 2022). It occurs when reactive oxygen species (ROS) attack polyunsaturated fatty acids (PUFAs) in cellular membranes. ROS, highly reactive molecules derived from molecular oxygen, include superoxide anions, hydrogen peroxide, and hydroxyl radicals. They are primarily generated through enzymatic reactions involving NADPH oxidases (NOXs), cytochrome P450 reductase (POR), and NADH-cytochrome b5 reductase (CYB5R1), with the mitochondrial electron transport chain (mETC) serving as another significant source (Ai et al., 2021; Yan et al., 2021). PUFAs, with their multiple double bonds, are particularly vulnerable to oxidative attack, leading to the formation of lipid peroxidation products (L-OOH) such as malondialdehyde (MDA) and 4-hydroxy-trans-2-nonenal (4-HNE) (Barrera et al., 2015). These toxic byproducts compromise membrane integrity, increase permeability, cause leakage of intracellular components, and trigger inflammatory responses, exacerbating cellular damage (Yang and Stockwell, 2016). Additionally, iron plays a critical role as a cofactor in the biosynthesis of lipoxygenases (LOXs) and cytochrome P450 enzymes, which are central to the generation of ROS during ferroptosis (Zou et al., 2020). Iron metabolism promotes lipid peroxidation through the Fenton reaction (Fe^2^⁺ + H₂O₂ → Fe³⁺ + ⋅OH + OH⁻), which produces hydroxyl radicals that amplify oxidative stress and accelerate lipid peroxidation, as well as the Haber–Weiss reaction (Dixon et al., 2012).

ROS-driven lipid peroxidation proceeds through three main stages: initiation, propagation, and termination. ROS, such as hydroxyl radicals, abstract allylic hydrogen atoms from PUFAs during initiation, generating lipid radicals (L⋅). In tell as the Haber–Weiss reaction (Dixon et al., 2012), adjacent lipids produce more lipid radicals and lipid hydroperoxides (L-OOH), thus sustaining the chain reaction (Hermetter et al., 2012; Volinsky and Kinnunen, 2013; Que et al., 2018). This process leads to severe oxidative damage if left unchecked, compromising membrane integrity and ultimately triggering ferroptosis.

Notably, the specific composition of membrane phospholipids, particularly the levels of phosphatidylethanolamine (PE) containing polyunsaturated fatty acids (PE-PUFAs), plays a crucial role in determining ferroptosis sensitivity (Kagan et al., 2017). Higher PE-PUFA content increases the susceptibility of cells to lipid peroxidation, thereby elevating the risk of ferroptosis (Kagan et al., 2017). Acyl-CoA synthetase long-chain family member 4 (ACSL4) catalyzes the conversion of PUFAs into PUFA-CoA derivatives, which are then incorporated into PE by lysophosphatidylcholine acyltransferase 3 (LPCAT3) (Kagan et al., 2017). This process enhances membrane susceptibility to oxidative damage, accelerating lipid peroxidation and ferroptosis.

However, lipid peroxidation is not entirely uncontrollable, as multiple regulatory mechanisms exist within cells. Glutathione peroxidase 4 (GPX4), a key inhibitor of ferroptosis, uses glutathione (GSH) as a cofactor to reduce phospholipid hydroperoxides to their corresponding alcohols, thereby protecting cells and membranes from oxidative damage. In addition to the pivotal role of GPX4, recent studies have identified several GPX4-independent pathways that regulate lipid peroxidation and ferroptosis, providing multilayered protection against oxidative stress. Membrane-bound O-acyltransferase 1/2 (MBOAT1/2) is a ferroptosis suppressor (Liang et al., 2023). As a member of the lysophospholipid acyltransferase (LPLAT) family, MBOAT1/2 transfers monounsaturated fatty acids (MUFA) from MUFA-CoA to lysophosphatidylethanolamine (lyso-PE), thereby competitively reducing the incorporation of PUFA-CoA into lyso-PE (Liang et al., 2023). This modification alters the cellular phospholipid profile by increasing PE-MUFA levels while decreasing PE-PUFA levels, the latter being the preferred substrate for lipid peroxidation and a key determinant of ferroptosis sensitivity (Liang et al., 2023). Thus, this selective remodeling decreases the pool of peroxidation-prone phospholipids, offering an alternative pathway to suppress ferroptosis independently of GPX4 and FSP1 (Liang et al., 2023). Moreover, iPLA2β, a member of the calcium-independent phospholipase A2 family, functions independently of glutathione by cleaving oxidized fatty acid chains from phospholipids, directly removing peroxidized products, effectively detoxifying damaged lipids, and maintaining membrane integrity (Chen et al., 2021). This activity is particularly crucial under conditions where GPX4 is absent or impaired, significantly suppressing lipid peroxidation and ferroptosis. Studies have also shown that iPLA2β reduces oxidized phospholipid levels mediated by ALOX12, thereby limiting the accumulation of lipid peroxidation products (Chen et al., 2021). This detoxification mechanism works synergistically with the lipid remodeling functions of MBOAT1 and MBOAT2, forming a multi-layered defense system against ferroptosis.

In summary, lipid peroxidation is central in ferroptosis, driven by ROS-mediated oxidative damage to PUFAs in cellular membranes. While GPX4 is a key regulator in detoxifying lipid peroxides, several GPX4-independent mechanisms, such as lipid remodeling by MBOAT1 and MBOAT2 and lipid detoxification by iPLA2β, provide additional layers of protection. These interconnected mechanisms further illuminate the complexity of ferroptosis regulation and underscore the essential role of lipid peroxidation in its execution.

#### 2.1.3 Collapse of antioxidant defense mechanisms

The antioxidant defense System in cells is critically dependent on glutathione peroxidase 4 (GPX4), a crucial metabolic enzyme that uses glutathione (GSH) to convert lipid peroxides into non-toxic alcohols, thereby minimizing harmful lipid peroxidation and safeguarding cells from ferroptosis. The activity of GPX4 is highly reliant on sufficient intracellular GSH levels, which are synthesized via the cystine/glutamate antiporter System Xc^−^. Comprising the subunits solute carrier family seven member 11 (SLC7A11) and solute carrier family three member 2 (SLC3A2), System Xc^−^ is a vital antioxidant System, facilitating the 1:1 exchange of cystine and glutamate across the cell membrane (Dixon et al., 2014). The imported cystine is reduced to cysteine within the cell, contributing to GSH synthesis (Dixon et al., 2014). GPX4 converts GSH to oxidized glutathione while reducing toxic lipid peroxides (L-OOH) to their corresponding alcohols (L-OH), thus lowering ROS production (Yang and Stockwell, 2016; Zheng and Conrad, 2020). Under normal physiological conditions, reduced GSH is present in concentrations 10 to 100 times greater than oxidized GSH (GSSG), making the GSH/GSSG ratio a common measure of cellular oxidative stress (Em et al., 2013; Lei et al., 2015; Hambright et al., 2017; Tang et al., 2021). Therefore, maintaining adequate GSH levels and GPX4 activity is crucial for cellular antioxidant defense; any disruption to this mechanism can lead to increased oxidative stress and subsequent cellular damage.

The function of GPX4 is precisely regulated by nuclear factor E2-related factor 2 (NRF2), a key transcription factor that activates the expression of various antioxidant genes, including those encoding GSH synthetase and superoxide dismutase (SOD), facilitating the clearance of excess ROS (Xie et al., 2016; Tang et al., 2021). Under normal conditions, NRF2 is tightly suppressed by Kelch-like ECH-associated protein 1 (KEAP1), which mediates NRF2 degradation through ubiquitination (Kobayashi et al., 2004). However, under oxidative stress, KEAP1 undergoes a conformational change that relieves its suppression, allowing NRF2 to stabilize and upregulate antioxidant gene expression (Sun et al., 2016).

In addition to GPX4 and NRF2, cells employ various antioxidant mechanisms, including SOD, catalase (CAT), and vitamins C and E, to combat oxidative stress (Chen et al., 2016). SOD effectively catalyzes the conversion of superoxide radicals into water and oxygen, serving as the first line of antioxidant defense, while CAT further decomposes hydrogen peroxide to prevent harmful hydroxyl radical formation (Yuewei et al., 2014; Baker et al., 2023). Vitamins C and E enhance cellular antioxidant capacity through their hydrophilic and lipophilic properties, respectively (Tm et al., 2019; Hu Q et al., 2021). However, inflammatory factors such as interleukin-1β (IL-1β) and tumor necrosis factor-alpha (TNF-α) can significantly inhibit SOD and CAT activity, reduce levels of vitamins C and E, and activate the nuclear factor-kappa B (NF-κB) pathway, exacerbating oxidative stress and stimulating inflammatory responses, thereby creating a vicious cycle (Ueda and Takasawa, 2018).

Additionally, the NADPH/FSP1/CoQ10 pathway, through the action of FSP1, reduces CoQ10 with NADPH, further inhibiting lipid peroxidation and ferroptosis (Frei et al., 1990). Research also indicates that the gtp cyclohydrolase 1/Tetrahydrobiopterin/dihydrofolate reductase (GCH1/BH4/DHFR) pathway, particularly DHFR, suppresses ferroptosis by promoting BH4 synthesis; inhibiting DHFR leads to decreased BH4 production, thereby enhancing lipid peroxidation (Soula et al., 2020; Fanet et al., 2021).

Mitochondria, as central organelles for iron, calcium, lipid, and amino acid metabolism, have limited iron content. However, elevated iron levels can disrupt mitochondrial iron homeostasis (Beckman and Ames, 1998). Research has demonstrated that inhibiting mitochondrial tricarboxylic acid cycles or electron transport chains can lower mitochondrial membrane hyperpolarization and lipid peroxidation, underscoring the critical role of mitochondrial pathways in ferroptosis (Steiner and Lang, 2017; Haileselassie et al., 2019).

In summary, the ferroptosis-related biological components discussed above are presented in Table 1 and Figure 1. Figure 1 was created using Adobe Illustrator 2019 (Adobe Inc., San Jose, CA, USA). The design of the signaling pathways related to iron metabolism, the Fenton reaction, lipid peroxidation, and antioxidant pathways was inspired by the overall concepts and graphical elements from the literature to ensure scientific accuracy and rigor (An et al., 2023).

**TABLE 1:** Ferroptosis-related biological components in osteoarthritis.

Biological components	Mechanism or intervention target	Function	Reference
TfR1	Cellular iron uptake	Mediates endocytosis of transferrin-bound iron	Aisen et al. (1978), Johnsen et al. (2019)
DMT1	Iron transmembrane transport	Transports iron ions into the cytoplasm	Pantopoulos (2004), Feng et al. (2020), Koike et al. (2020)
STEAP3	Iron reduction	Reduces Fe3^+^ to Fe2^+^ for cytoplasmic iron pool	Pantopoulos (2004), Feng et al. (2020), Koike et al. (2020)
FPN1	Transmembrane iron export	Exports excess iron out of the cell	Kakhlon and Cabantchik (2002)
NCOA4	Ferritin autophagy	Regulates intracellular iron levels by ferritin degradation	Latunde-Dada (2017)
GPX4	Antioxidant enzyme activity	Protects cells from oxidative damage and ferroptosis by reducing lipid peroxides	Mancias et al. (2014)
System Xc^-^	Cystine/glutamate exchange	Mediate’s cystine uptake for GSH synthesis	Dixon et al. (2014)
NRF2	Antioxidant gene expression	Regulates antioxidant gene expression under oxidative stress	Xie et al. (2016), Tang et al. (2021)
SOD	Superoxide radicals	Catalyzes the dismutation of superoxide radicals	Cheng et al. (2022)
CAT	Hydrogen peroxide	Decomposes hydrogen peroxide to water and oxygen	Baker et al. (2023)
NADPH	CoQ10 reduction	Reduces CoQ10 to inhibit lipid peroxidation	Frei et al. (1990)
FSP1	NADPH-dependent CoQ10 reduction	Catalyzes NADPH reduction of CoQ10	Frei et al. (1990)
GCH1	BH4 synthesis	Promotes BH4 synthesis to inhibit ferroptosis	Soula et al. (2020), Fanet et al. (2021)
DHFR	BH4 synthesis	Promotes BH4 synthesis, reducing lipid peroxidation	Soula et al. (2020), Fanet et al. (2021)
ACSL4	Promotes lipid peroxidation	Accelerates lipid peroxidation	Kagan et al. (2017)

## 3 The role of ferroptosis in osteoarthritis

Osteoarthritis (OA) is characterized by pathological changes affecting the entire joint structure, with degenerative alterations in articular cartilage being the primary manifestation (Findlay and Atkins, 2014). These pathological changes are accompanied by bone spur formation, subchondral bone sclerosis, and synovitis. Under abnormal biomechanical and biochemical stimuli, the balance between anabolic and catabolic processes in chondrocytes is disrupted, leading to the characteristic phenotypic changes associated with OA and promoting disease progression. For example, hypertrophic chondrocytes release matrix degradation products and pro-inflammatory mediators, which aggravate cartilage damage and trigger the proliferation of nearby synovial cells and inflammatory responses (Hunter and Bierma-Zeinstra, 2019; Singh et al., 2019). In turn, these proliferating synovial cells release pro-inflammatory mediators that stimulate the expression of matrix-degrading enzymes and other catabolic factors (Hunter and Bierma-Zeinstra, 2019; Singh et al., 2019). The formation of bone spurs at the joint margins is closely linked to cartilage formation and ossification, a process influenced by inflammatory factors and joint overload (Hsia et al., 2018). Additionally, changes in subchondral bone contribute to cartilage destruction, primarily through an imbalance between osteoblastic and osteoclastic activity (Hu Y et al., 2021).

**FIGURE 2:**
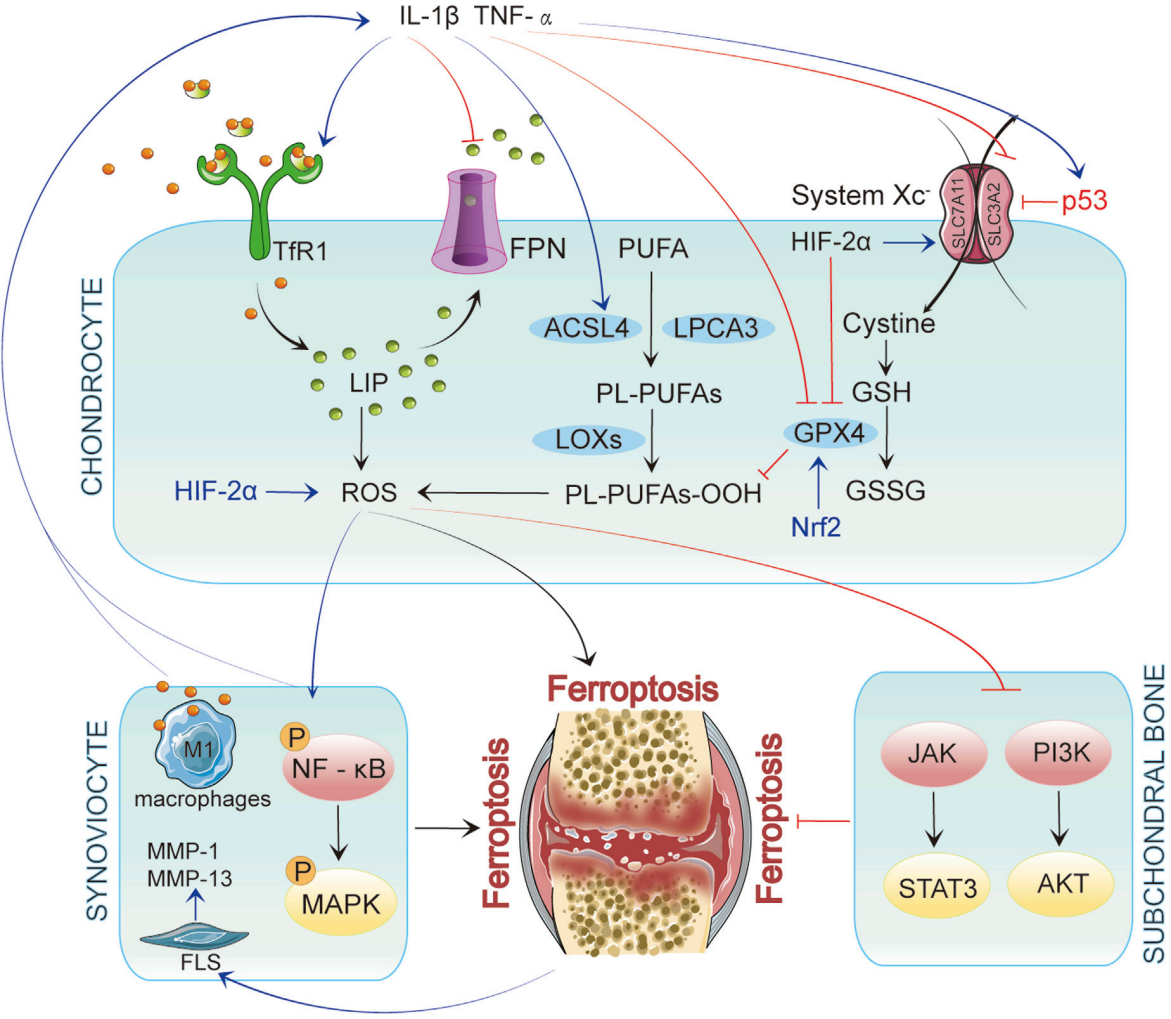
In Chondrocytes, ferroptosis is initiated by iron accumulation and lipid peroxidation. When p53 is upregulated, it inhibits System Xc^−^, leading to decreased glutathione levels and reduced GPX4 activity, ultimately resulting in ferroptosis. Inflammatory factors such as IL-1β and TNF-α enhance the generation of reactive oxygen species (ROS) by activating TfR1 and ACSL4 while upregulating p53 expression, further promoting ferroptosis. These inflammatory mediators affect chondrocytes and interact with synovial cells and their microenvironment, exacerbating inflammation in OA. In synovial cells, inflammatory signaling pathways activate NF-κB and MAPK, promoting ferroptosis. Concurrently, iron overload polarizes synovial macrophages to the classical M1 type, leading to increased secretion of inflammatory mediators. This activation of inflammation further drives ferroptosis, which activates fibroblast-like synoviocytes (FLSs) to produce matrix metalloproteinases (MMP-1 and MMP-13), intensifying synovitis and creating a vicious cycle. In subchondral bone, ferroptosis is similarly regulated by multiple signaling pathways, particularly the JAK/STAT3 and PI3K/AKT axes.

Recent studies have identified iron overload as a risk factor for osteoarthritis. While research on the relationship between ferroptosis and OA is limited, emerging evidence suggests that ferroptosis plays a crucial role in regulating chondrocyte activity, extracellular matrix degradation, and synovial inflammation, thereby influencing OA progression (Feng et al., 2020; Yao et al., 2021). Elevated iron levels in OA patients’ joint tissues may trigger chondrocyte ferroptosis by enhancing oxidative stress and lipid peroxidation (Feng et al., 2020). In elderly female OA patients, higher iron storage levels may be associated with increased iron burden due to estrogen deficiency. Patients with significantly elevated ferritin levels are more prone to severe joint complications, and this increase correlates positively with the severity of arthritis (Morales-Ivorra et al., 2018; Jing et al., 2021a). However, some studies propose a biphasic effect of iron status on OA progression, where iron overload and deficiency increase OA risk (Kennish et al., 2014). This article will explore how ferroptosis impacts various pathological processes in OA, aiming to provide new insights into its prevention and treatment (Figure 2). Figure 2 was created using Adobe Illustrator 2019 (Adobe Inc., San Jose, CA, USA).

### 3.1 Cartilage degeneration and ferroptosis

A prominent feature of OA is the degeneration and degradation of articular cartilage, closely linked to the metabolic balance of chondrocytes (Zheng et al., 2021). Chondrocytes, the only cellular component of cartilage, maintain the dynamic equilibrium of the extracellular matrix (ECM) through precise regulation of collagen type II and matrix-degrading enzyme secretion (Chang et al., 2022). However, this balance is disrupted during the pathological progression of OA, leading to metabolic imbalances in chondrocytes and consequent cartilage degradation. Ferroptosis is crucial in the loss of chondrocytes associated with this process.

In mouse models of OA, typical characteristics of ferroptosis have been observed in chondrocytes, including abnormal iron metabolism, lipid peroxidation, and mitochondrial dysfunction (Sun et al., 2021). These alterations impair normal chondrocyte function and exacerbate apoptosis and ECM degradation (Sun et al., 2021). Under iron overload conditions, catabolic pathways in chondrocytes are activated, while anabolic pathways are suppressed, destroying the cartilage matrix and progression of OA. In OA models induced by interleukin-1 beta (IL-1β) and ferric ammonium citrate (FAC), hallmark ferroptosis markers such as glutathione peroxidase 4 (GPX4) and solute carrier family seven member 11 (SLC7A11) exhibit significant downregulation, whereas expression levels of the tumor suppressor gene p53 and acyl-CoA synthetase long-chain family member 4 (ACSL4) are elevated (Yao et al., 2021). These findings further corroborate the involvement of ferroptosis in chondrocytes, indicating mechanisms related to lipid peroxidation and oxidative stress imbalance.

Recent studies have highlighted the mediating role of hypoxia-inducible factor 2 alpha (HIF-2α) in chondrocyte ferroptosis, promoting lipid storage and accumulation of lipid peroxidation products. Overexpression of HIF-2α decreases GPX4 levels and facilitates the activity of carnitine palmitoyltransferase 1A (CPT1A), which transports fatty acids into the mitochondrial matrix for β-oxidation, thus aggravating cartilage degeneration (Zhou et al., 2021).

Moreover, inflammatory cytokines have been found to disrupt iron homeostasis in chondrocytes by upregulating transferrin receptor 1 (TfR1) and downregulating the iron transporter ferroportin (FPN), contributing to iron overload (Jing et al., 2020). In OA, This iron overload is closely related to disease progression, as it not only directly induces chondrocyte apoptosis but also accelerates cartilage degradation by upregulating matrix-degrading enzyme expression (Jing et al., 2021a). For instance, iron overload induced by ferric ammonium citrate significantly enhances chondrocyte apoptosis and increases the expression of matrix metalloproteinases (MMP3, MMP13) (Jing et al., 2021a). Furthermore, downregulation of nuclear factor E2-related factor 2 (Nrf2) leads to reduced GPX4 expression, thereby intensifying ferroptosis (Ni et al., 2020; Lu et al., 2022; Yang et al., 2022b). As a key antioxidant transcription factor, Nrf2’s interaction with ferroptosis illustrates its critical role in regulating oxidative stress and cellular fate.

Beyond directly disrupting iron homeostasis, ferroptosis further contributes to the pathological progression of OA through additional mechanisms. The roles of HIF-2α, GPX4, and lipid peroxidation in ferroptosis are particularly critical. Ferroptosis damages chondrocytes directly and exacerbates joint injury by inducing local inflammatory responses. For instance, iron overload can enhance chondrocyte catabolism through overactivated NLRP3 inflammasomes, promoting MMP expression and exacerbating cartilage degeneration (Li S et al., 2023).

Recent studies have established a close relationship between ferroptosis and ECM degradation (Wilkinson et al., 2019). The ECM, composed of proteoglycans, collagen, and minerals, is essential for maintaining the biomechanical properties of articular cartilage. However, as chondrocyte function declines, tissue hydration decreases, leading to progressive ECM damage. In the early stages of OA, proliferating chondrocytes disrupt ECM composition, resulting in reduced proteoglycan synthesis and altered matrix permeability and mechanical compliance (Sun et al., 2012). Inflammatory factors and proteases play pivotal roles in ECM degradation. Evidence suggests that iron regulates ECM deposition and remodeling via oxidative stress, indicating its critical role in ECM integrity (Maldonado and Nam, 2013; Wilkinson et al., 2019). Additionally, mechanical injuries, such as joint wear, trigger inflammatory responses in cartilage and synovium, further upregulating the expression of matrix-degrading proteins, accelerating ECM degradation, and promoting OA progression (Maldonado and Nam, 2013). Studies have shown that iron can enhance IL-1β-induced expression of MMP-3 and MMP-13, further facilitating ECM degradation (Maldonado and Nam, 2013). This association with iron accumulation in chondrocytes is characterized by IL-1β upregulating TfR1 and DMT1 while downregulating FPN1, which exacerbates iron overload, disrupts ECM balance, and ultimately leads to cartilage damage (Jing et al., 2020; Jing et al., 2021). While the negative impact of iron overload on ECM has been established, the specific mechanisms by which ferroptosis contributes to ECM degradation remain underexplored. Given that ECM degradation involves inflammation and oxidative stress—key components of ferroptosis—a complex relationship between ferroptosis and ECM degradation warrants further investigation.

In recent years, the roles of oxidative stress and mitochondrial dysfunction in the progression of OA have garnered significant attention (Blanco et al., 2011; Suantawee et al., 2013; Ansari et al., 2020; Wang et al., 2020; Zheng et al., 2021). Excessive iron can induce mitochondrial dysfunction in chondrocytes by producing reactive oxygen species (ROS), thereby promoting the expression of OA catabolic markers (Jing et al., 2021a). However, the implications of iron-induced mitochondrial dysfunction on chondrocyte metabolism require deeper exploration (Jing et al., 2021b). Additionally, studies suggest that the mitochondrial membrane potential in chondrocytes decreases significantly impacts mitochondrial structure and function (Jing et al., 2021b). Under IL-1β stimulation, chondrocyte mitochondrial membrane potential decreases substantially, closely correlating with mitochondrial dysfunction (Chen B. Y et al., 2024). The interconnections among ferroptosis, oxidative stress, and mitochondrial dysfunction in OA cartilage degeneration collectively influence chondrocyte metabolism, ECM balance, and the progression of OA. Nonetheless, these three elements’ specific regulatory networks and mechanisms require further elucidation.

### 3.2 Synovial inflammation and ferroptosis

Synovial inflammation is another critical feature in the pathological progression of OA and may even precede cartilage degeneration (Scanzello and Goldring, 2012). Synovial changes are closely linked to symptoms such as pain and functional impairment. Research suggests that iron homeostasis in synovial tissue is regulated by iron regulatory proteins, revealing a strong link between synovitis, synovial hyperplasia, and iron deposition (Nieuwenhuizen et al., 2013). For instance, in patients with hemophilia, significant deposits of hemosiderin in the periosteum can stimulate fibroblast proliferation, leading to hemophilic synovitis (Wen et al., 2002; Nieuwenhuizen et al., 2013). Synovial lesions mainly impact areas near cartilage degeneration and are marked by synovial lining cell hyperplasia, infiltration of inflammatory cells, neovascularization, and fibrin deposition (Sellam and Berenbaum, 2010).

Recent research progressively highlights the potential role of ferroptosis in synovial cells as a major factor contributing to synovial inflammation. Compared to healthy individuals, OA patients exhibit significantly elevated levels of iron ions in synovial fluid, alongside observable iron deposits in synovial tissue (Yazar et al., 2005). Under conditions of iron overload, macrophages in the synovium polarize into classically activated M1 macrophages (Hakobyan et al., 2004; Nieuwenhuizen et al., 2013; Zhou et al., 2018; Wu et al., 2020). This process is closely associated with ROS production induced by iron overload and p53 acetylation (Stockwell et al., 2017). M1 macrophages secrete pro-inflammatory cytokines such as IL-1β, IL-6, and TNF-α, which trigger further inflammatory responses in the synovium and contribute to cartilage destruction and osteophyte formation, thus exacerbating OA progression (Marchev et al., 2017; Ansari et al., 2020). Ferroptosis induced by iron overload may also disrupt the synovial microenvironment, impairing the function of fibroblast-like synoviocytes (FLS). Ferroptosis can trigger a non-specific immune response, leading to the release of IL-1β, which activates FLS to produce MMP-1 and MMP-13—proteins closely associated with synovial inflammatory responses (Ni et al., 2018; Jing et al., 2021a). Additionally, IL-1β promotes the expression of cyclooxygenase-2 (COX-2) in FLS, enhancing the secretion of prostaglandin E2 (PGE2) and further aggravating synovial inflammation (Tsai et al., 2017).

The release of lipid peroxidation products and oxidative stress signals during ferroptosis activates inflammatory pathways in synovial cells, such as the NF-κB and MAPK pathways, resulting in the release of additional inflammatory factors and creating a vicious cycle (Kumar et al., 2019; Tm et al., 2019; Liu et al., 2022; Lv et al., 2022; Miao et al., 2022). ACSL4 is highly expressed in OA synovial tissue, while the key antioxidant enzyme GPX4 expression is significantly reduced. ACSL4 influences the oxidative-antioxidative balance within cells, promoting ferroptosis, whereas the deficiency of GPX4 exacerbates ferroptosis and associated inflammatory responses (He W et al., 2023; Zheng et al., 2024).

Moreover, an imbalance in the exchange of glutamate and cystine in the System Xc^−^ transport system inhibits its function, leading to glutathione depletion and indirect suppression of GPX4 activity, thus facilitating ferroptosis. Glutamate may also induce intra-articular inflammatory responses and abnormal pain in OA patients by activating N-methyl-D-aspartic acid (NMDA) receptors, further advancing OA progression (Piepoli et al., 2009).

### 3.3 Subchondral bone remodeling and ferroptosis

Subchondral bone remodeling is a hallmark pathological feature in advanced stages of OA, typically characterized by bone sclerosis and osteophyte formation. Research indicates a close interplay between articular cartilage and subchondral bone. On the one hand, abnormal remodeling and angiogenesis in subchondral bone can destroy articular cartilage. On the other hand, chondrocytes can regulate subchondral bone remodeling through the RANK-RANKL-OPG signaling pathway (Hu Y et al., 2021). Mechanistically, the core of bone remodeling lies in the balance between osteoblasts’ bone-forming activity and osteoclasts’ bone-resorbing activity (Simao and Cancela, 2021). The death of chondrocytes and the cartilage matrix’s degradation can disrupt the cartilage-bone interface’s structure, consequently affecting osteoblasts’ and osteoclast activity (Miao et al., 2022). In this process, ferroptosis plays several critical roles (Miao et al., 2022).

Studies have shown that iron overload elevates intracellular ROS levels, triggering lipid peroxidation, disrupting redox balance, and altering the bone marrow microenvironment (Cen et al., 2018). This process blocks the JAK/STAT3 and PI3K/AKT signaling pathways, activates the p38-MAPK pathway, and induces G1 cell cycle arrest and autophagy in osteoblasts (Cen et al., 2018). Elevated ROS also promotes osteoclast differentiation via NF-κB, JNK, and ERK signaling, driving abnormal bone remodeling and OA progression (Wang et al., 2018). Furthermore, iron overload can directly impair osteoblasts’ and osteoclast functions and alter the bone matrix’s composition and structure, facilitating bone sclerosis and osteophyte formation. This abnormal bone remodeling further increases the mechanical burden on the joint, exacerbating OA symptoms (Hu Y et al., 2021).

Additionally, hypoxia-inducible factor 1 (HIF-1) is a key regulator of chondrocyte growth and survival, significantly influencing the state of subchondral bone. Research indicates that HIF-1α-induced long non-coding RNA (lncRNA) PMAN can regulate ferroptosis by maintaining the mRNA stability of the ferroptosis-related gene SLC7A11, inhibiting ferroptosis and contributing to subchondral bone remodeling (Fan et al., 2018; Lin et al., 2022). This interaction affects the progression of osteoarthritis. However, the specific roles of ferroptosis-related genes and miRNAs remain to be explored further, representing a potential direction for future research into ferroptosis.

In conclusion, the biological molecules of ferroptosis involved in this part are shown in Tables 1, 2.

**TABLE 2:** Ferroptosis-related biological components in osteoarthritis.

Biological components	Intervention target	Function	Reference
SLC7A11	Antioxidant enzyme system	Inhibits oxidative stress and lipid peroxidation	Yao et al. (2021)
CPT1A	Facilitates fatty acid β-oxidation	Increases lipid oxidation	Zhou et al. (2021)
MMP-1	synovial	Related to synovitis and inflammation	Ni et al. (2018), Jing et al. (2021a)
MMP-13	synovial	Related to synovitis and inflammation	Ni et al. (2018), Jing et al. (2021a)
COX-2	arachidonic acid	Increases prostaglandin E2 secretion	Tsai et al. (2017)
NMDA receptor	OA joint	Triggers inflammation and pain in OA progression	Piepoli et al. (2009)
ROS	Osteoblasts and Osteoclasts	Disrupts bone cell function, leading to abnormal bone remodeling	Stockwell et al. (2017)
HIF-1α	Cartilage Cells (Chondrocytes)	Critical for the growth and survival of chondrocytes, influencing subchondral bone	Fan et al. (2018), Lin et al. (2022)
LncRNA PMAN	SLC7A11 mRNA	Maintains the stability of ferroptosis-regulating gene SLC7A11 mRNA, inhibiting ferroptosis	Fan et al. (2018), Lin et al. (2022)
FOXO3	NF-κB/MAPK signaling pathway	Upregulates antioxidant genes, reduces lipid peroxidation and iron accumulation, protects chondrocytes and ECM	Zhao et al. (2023)
BoNT/A	SLC7A11/GPX4 anti-ferroptosis system	Reduces ROS and iron ion accumulation, restores mitochondrial function	Zeng et al. (2024)

## 4 Biomarkers related to ferroptosis and clinical prospects in osteoarthritis

Progressive joint pain is a hallmark of osteoarthritis (OA), and treatments aim to alleviate pain and improve joint function. Conventional treatments encompass lifestyle changes, physical therapy, medication, and surgical interventions, with effectiveness depending on disease severity and duration, highlighting the need for individualized treatment. New therapies such as nerve blocks, mesenchymal stem cell injections, platelet-rich plasma injections, and osteoporosis medications like strontium ranelate offer additional options for OA management. However, due to the incomplete understanding of OA’s pathogenesis, recognized effective treatment methods remain limited.

Iron homeostasis imbalance, leading to iron overload and subsequent ferroptosis, can damage the synovium, cartilage, and extracellular matrix (ECM), playing a crucial role in OA progression. Key proteins and genes involved in iron metabolism, lipid peroxidation, and associated signaling pathways may be potential therapeutic targets for OA. Ferroptosis-related molecular biomarkers hold great potential for diagnosing OA, monitoring disease progression, and guiding personalized treatment strategies. The following sections will delve into biomarkers associated with ferroptosis, iron metabolism, lipid peroxidation, and antioxidant defenses, analyzing their clinical application prospects in OA.

### 4.1 Iron metabolism-related biomarkers and osteoarthritis

#### 4.1.1 Transferrin receptor 1 (TfR1)

TfR1 expression is elevated in OA patients’ synovial tissue and chondrocytes, making it a potential early diagnostic marker that reflects disease progression. Its integration with imaging techniques can enhance diagnostic accuracy, facilitating timely intervention. Research on TfR1 has identified several therapeutic interventions. For example, ferristatin II promotes the degradation of TfR1, disrupting Tf-mediated iron delivery and intervening in the pathological processes of OA (Yuewei et al., 2014). Additionally, Biochanin A (BCA) can reduce intracellular iron levels by suppressing TfR1 activity and enhancing the expression of ferroportin (FPN), thereby alleviating mitochondrial damage and chondrocyte apoptosis induced by iron overload (He Q et al., 2023).

Iron chelators represent an effective intervention for patients with excessive TfR1 expression, binding circulating and intracellular iron for excretion via urine or bile, thereby reducing ferroptosis (Di Maggio and Maggio, 2017). Commonly used iron chelators include deferoxamine (DFO), deferiprone (DFP), and deferasirox (DFS) (Rodrigues de Morais and Gambero, 2019). *In vitro* studies have demonstrated that DFO can inhibit the degradation of type II collagen in osteoarthritis cartilage while also reducing the expression of inflammatory factors such as MMP-1, MMP-13, IL-1β, and TNF-α (Tchetina et al., 2016). Furthermore, DFO can restore ferritin levels by inhibiting NCOA4 expression and chelating excess iron to regulate iron homeostasis. These findings indicate that iron chelators regulate iron levels and contribute to managing inflammation associated with OA.

However, existing iron chelators exhibit side effects in clinical applications, including gastrointestinal discomfort, hepatotoxicity, and nephrotoxicity (Guo et al., 2024). Thus, developing novel iron chelators, particularly nanoscale formulations, may represent a future direction to reduce side effects while maintaining or enhancing therapeutic efficacy (Kalanaky et al., 2016). Additionally, the compound White Cardamonin (CAR), derived from Vitex negundo, protects cartilage by mitigating iron overload-induced chondrocyte damage and apoptosis (Li S et al., 2023). CAR activates the SIRT1 pathway and inhibits the p38 MAPK signaling pathway, reducing NLRP3 inflammasome production and alleviating inflammation and cartilage degeneration (Li S et al., 2023). It also reverses the reduction in type II collagen expression and the elevation of MMPs caused by iron overload, thereby mitigating chondrocyte degeneration (Li S et al., 2023).

Based on TfR1-mediated iron uptake and ferroptosis mechanisms, researchers are exploring possibly delaying OA progression by intervening in ferroptosis. Inhibiting TfR1 expression may reduce chondrocyte ferroptosis and diminish synovial inflammation, slowing OA advancement. Currently, ferroptosis inhibitors such as Ferrostatin-1 and Liproxstatin-1 have entered clinical trials, potentially leading to precision treatment strategies that integrate TfR1 detection techniques (Li S et al., 2023).

Despite the promising clinical prospects of TfR1 in OA, challenges remain in its practical application. Future research should further validate the specificity and sensitivity of TfR1 as an early diagnostic marker and develop cost-effective detection technologies. Moreover, multi-omics analyses could help elucidate the interactions between TfR1 and other ferroptosis regulatory molecules, such as GPX4, providing a comprehensive perspective to support personalized treatment strategies.

#### 4.1.2 Divalent metal ion transporter 1 (DMT1)

DMT1 plays a crucial role in the transport of iron ions, and its abnormal expression in OA patients is closely associated with disease progression. Research has shown that the upregulation of DMT1 results in excessive iron accumulation within chondrocytes, increasing the risk of cellular damage (Li S et al., 2023). Research indicates that DMT1 is critical in the progression of osteoarthritis (OA) driven by iron overload; inhibiting DMT1 alleviates IL-1β-induced inflammatory responses and extracellular matrix degradation by significantly suppressing the MAPK and PI3K/AKT/NF-κB signaling pathways (Liu et al., 2024). This finding suggests that regulating the activity of the iron transporter DMT1 could offer a novel therapeutic target for OA.

Implementing DMT1 inhibitors as an intervention strategy demonstrates significant therapeutic potential (Jing et al., 2020). Additionally, multi-target combination strategies are gaining attention. For instance, combining DMT1 inhibitors with antioxidants can yield synergistic effects across multiple pathways. This approach reduces iron ion accumulation, suppresses ferroptosis, and enhances antioxidant defense mechanisms, thereby protecting chondrocytes from oxidative stress damage and effectively slowing OA progression.

#### 4.1.3 Ferritin

Ferritin consists of heavy chain ferritin (FTH1) and light chain ferritin (FTL), and it primarily functions to store intracellular iron, preventing excessive accumulation of free iron and reducing cellular iron toxicity (Arosio et al., 2017). In OA patients, alterations in ferritin expression and stability lead to elevated levels of free iron, heightening the risk of ferroptosis. Studies have shown that in models of iron overload and destabilization of the medial meniscus (DMM)-induced OA, FTH1 expression is significantly elevated, accompanied by increased ferritin levels in the synovial tissue (Yuan et al., 2024). Thus, monitoring changes in both FTH1 and FTL may provide new insights for early OA diagnosis.

Combining the detection of ferritin with other ferroptosis biomarkers, such as TfR1, DMT1, and GPX4, holds promise for enhancing diagnostic sensitivity and accuracy, thereby offering stronger support for early intervention. Additionally, variations in ferritin levels may serve as auxiliary indicators for evaluating the efficacy of antioxidant treatments (Arosio et al., 2017). The concurrent use of antioxidants, such as N-acetylcysteine (NAC), may improve treatment precision and reduce adverse effects (de Andrade et al., 2015; Li et al., 2022).

Furthermore, ferritin plays a dual role in iron metabolism imbalance; while it helps sequester excess free iron, it may also act as a source of free iron during ferroptosis (Bauckman et al., 2015; Bellelli et al., 2016). Research has identified YL-939, a ferroptosis inhibitor targeting prohibitin 2 (PHB2), which can reduce iron levels in the labile iron pool (LIP) by modulating ferritin expression and uptake (Yang et al., 2022a). Consequently, in future developments of ferroptosis inhibitors, ferritin is anticipated to be a potential target. Its combination with iron chelators and agents that regulate ferritin could further enhance intervention strategies for ferroptosis, providing new avenues for OA treatment.

#### 4.1.4 Ferroportin (FPN)

FPN is mammalian cells’ sole iron exporter, pivotal in maintaining intracellular iron homeostasis. Recent studies have highlighted the significance of FPN in regulating ferroptosis. The degradation of FPN, mediated by the E3 ubiquitin ligase RNF217, leads to intracellular iron accumulation, thereby increasing susceptibility to ferroptosis (Jiang et al., 2021). Therefore, maintaining proper FPN function is crucial for preventing iron overload and subsequent ferroptotic cell death in joint tissues.

Therapeutic approaches to prevent FPN degradation may hold promise for mitigating OA progression. For instance, targeting the RNF217-mediated degradation pathway of FPN could help preserve its function, thereby reducing intracellular iron accumulation and the risk of ferroptosis. Additionally, combining such approaches with antioxidants or ferroptosis inhibitors might synergize in protecting chondrocytes and synovial cells from iron-induced damage (Jiang et al., 2021).

Further research is needed to elucidate the precise role of FPN in OA and to develop targeted therapies that modulate its expression or activity. Understanding the interplay between iron metabolism, FPN regulation, and ferroptosis will be essential in devising effective interventions for OA and other iron-related pathologies.

#### 4.1.5 Hepcidin and NCOA4

Hepcidin and NCOA4 play crucial roles in regulating iron homeostasis and ferroptosis, and their abnormal expression is closely linked to the progression of OA (Bellelli et al., 2016; Wang and Babitt, 2019). Hepcidin, a central regulatory hormone in iron metabolism, promotes the degradation of FPN, leading to decreased extracellular iron export and subsequent intracellular iron overload (Wang and Babitt, 2019). This mechanism exacerbates chondrocyte damage associated with ferroptosis in OA patients. Concurrently, NCOA4 mediates the autophagy of ferritin, releasing stored iron and further increasing free iron levels within cells (Bellelli et al., 2016). This process induces oxidative stress and lipid peroxidation, accelerating ferroptosis and driving the progression of OA.

Targeting these mechanisms by inhibiting the activity of hepcidin and NCOA4 offers new therapeutic avenues for OA. Recently, a class of ferroptosis inhibitors that target NCOA4 has been identified. These compounds bind to NCOA4, disrupting its interaction with FTH1 and inhibiting NCOA4-dependent ferritin autophagy, thereby reducing intracellular free iron (Fe^2^⁺) levels and suppressing ferroptosis (Fang et al., 2021).

### 4.2 Lipid peroxidation-related biomarkers and OA

#### 4.2.1 Malondialdehyde (MDA) and 4-Hydroxynonenal (4-HNE)

MDA and 4-HNE are key end products of lipid peroxidation and significant markers of ferroptosis. Their levels are markedly elevated in OA patients’ synovial fluid, cartilage, and serum and positively correlate with disease severity. Thus, MDA and 4-HNE are considered effective biomarkers for reflecting the progression of OA (Shah et al., 2005; Mas-Bargues et al., 2021). Studies show that MDA levels significantly increase in IL-1β- or erastin-induced OA mouse models, while 4-HNE and its protein adducts rise in the synovial fluid and chondrocytes of OA patients, underscoring the critical role of lipid peroxidation in OA pathology (Grigolo et al., 2003; Shah et al., 2005; Gladkova, 2022).

Targeting this mechanism, antioxidants like astaxanthin exhibit potential therapeutic effects (Wang et al., 2022a). Astaxanthin mitigates the downregulation of ferroptosis-related proteins induced by IL-1β by downregulating p53 expression, effectively inhibiting chondrocyte ferroptosis (Wang et al., 2022a). Forkhead Box O3 (FOXO3), an important transcription factor, reduces lipid peroxidation and intracellular iron accumulation by upregulating antioxidant genes and inhibiting the NF-κB/MAPK signaling pathway, thereby lessening chondrocyte ferroptosis and ECM degradation (Zhao et al., 2023). *In vitro* experiments indicate that stimulating FOXO3 expression significantly decreases the generation of ferroptosis-related markers like MDA, suggesting its potential to suppress OA progression (Zhao et al., 2023).

Moreover, Tanshinone IIA (Tan IIA) can inhibit lipopolysaccharide (LPS)-induced iron hypersensitivity by lowering iron, reactive oxygen species, and MDA levels while increasing glutathione (GSH) levels (Xu J et al., 2023). These findings suggest that Tan IIA may demonstrate potential clinical value in OA treatment by improving oxidative stress conditions. Recent studies show that naringin (NAR), an active component of grapefruit, possesses significant anti-inflammatory and antioxidant properties (Pan et al., 2022). *In vitro*, NAR has been found to reduce intracellular MDA levels and ROS accumulation while upregulating the antioxidant gene NRF2, indicating its ability to alleviate oxidative stress and chondrocyte damage induced by iron overload (Pan et al., 2022).

As terminal products of lipid peroxidation, MDA and 4-HNE directly reflect the extent of lipid oxidative damage within cells. Therefore, monitoring these biomarkers can aid in the early diagnosis of OA and serve for disease monitoring and prognostic assessment, facilitating the development of more targeted treatment strategies.

#### 4.2.2 Glutathione peroxidase 4 (GPX4)

GPX4 is a crucial enzyme inhibiting ferroptosis, protecting cell membranes from oxidative damage by reducing lipid peroxides. A decline in GPX4 function directly increases the risk of ferroptosis, making its expression changes in OA a significant marker for assessing this risk. Clinically, monitoring GPX4 levels in articular cartilage can enhance the accuracy of early OA diagnosis.

Recent studies have explored therapeutic strategies targeting the GPX4 axis to suppress ferroptosis. For example, icariin (ICA) can upregulate GPX4 expression, reducing MDA levels and iron content in synovial cells, thereby activating the System Xc^−^/GPX4 axis to inhibit ferroptosis and alleviate synovial inflammation (Wang et al., 2016; Xiao et al., 2024). Such research opens possibilities for developing GPX4-based OA treatments (Xiao et al., 2024). Additionally, thiazolidinedione (TZD) drugs selectively inhibit ACSL4 activity, significantly reducing lipid peroxidation and ferroptosis (Doll et al., 2017).

Combining GPX4 with iron chelators, antioxidants, or free radical scavengers such as Fer-1, Lip-1, vitamin E, or edaravone can further enhance its protective effects, reducing lipid oxidative damage and slowing OA progression (Kagan et al., 2017; Homma et al., 2019). Studies indicate that in mild OA patients, using ferroptosis inhibitors like Ferrostatin-1 (Fer-1) can significantly upregulate GPX4 and SLC7A11 expression while downregulating ACSL4 expression, suggesting the potential for early intervention with these inhibitors (Y. Xu Y et al., 2023). However, this regulatory effect weakens in moderate to severe OA patients, indicating that early treatment may be more effective, with additional therapies needed in later stages for optimal results (Xu Y et al., 2023).

In clinical practice, selecting ferroptosis inhibitors or antioxidants based on GPX4 level changes can substantially improve patient outcomes. For patients with markedly reduced GPX4 expression, antioxidants like NAC could enhance antioxidant capacity and delay disease progression. Although GPX4’s potential in OA treatment is evident, further validation as a therapeutic target is necessary. Future research should explore GPX4’s specific roles in different OA subtypes, developing personalized treatments based on GPX4.

#### 4.2.3 Membrane-bound O-Acyltransferase 1/2 (MBOAT1/2)

MBOAT1/2, as critical membrane-bound O-acyltransferases, functions by incorporating monounsaturated fatty acids (MUFA) into phospholipids, thereby reducing polyunsaturated fatty acids (PUFA) levels. This mechanism decreases the proportion of peroxidation-prone phospholipids, thereby enhancing cellular resistance to ferroptosis. These enzymes have demonstrated potential therapeutic value in mitigating lipid peroxidation and ferroptosis (Liang et al., 2023). Studies have shown that these enzymes are transcriptionally regulated by sex hormone receptors—estrogen receptor (ER) and androgen receptor (AR)—which mediate ferroptosis surveillance independent of GPX4 and FSP1 (Liang et al., 2023).

Postmenopausal women experience a significant decline in estrogen levels, associated with an increased incidence of OA (Parazzini and Progretto Menopausa Italia Study Group, 2003; Herndon, 2004; Høegh-Andersen et al., 2004). Researchers using an ovariectomized rat model found that the decline in estrogen levels significantly increased the metabolic activity of subchondral bone, leading to frequent microdamage and remodeling. This process resulted in increased subchondral bone stiffness, which subsequently altered the local biomechanical environment of the joint, exacerbating cartilage damage and accelerating the progression of OA (Sowers et al., 1996; Wu et al., 2019; Xu et al., 2019; Ziemian et al., 2021). Given the pivotal role of MBOAT1/2 in suppressing oxidative stress and ferroptosis, strategies aimed at restoring their functions may offer therapeutic benefits in addressing joint damage associated with sex hormone deficiencies (Liang et al., 2023). For instance, hormone replacement therapy (HRT) or pharmacological agents mimicking estrogen may enhance the protective role of MBOAT1, thereby inhibiting ferroptosis and lipid peroxidation (Liang et al., 2023).

Targeted therapeutic strategies that regulate MBOAT1/2 activity, particularly for hormone-deficient populations, could serve as a promising approach to delay OA progression. Combining MBOAT1/2-enhancing interventions with antioxidants or ferroptosis inhibitors may yield synergistic effects. Future research should focus on elucidating the specific mechanisms of hormone-MBOAT1/2 interactions in OA treatment and further validating their clinical applications in personalized therapies.

#### 4.2.4 Lipid reactive oxygen species (Lipid-ROS)

Lipid-ROS are reactive oxygen species produced during lipid peroxidation, and their accumulation is a core feature of ferroptosis. In OA, elevated levels of Lipid-ROS are closely linked to chondrocyte death and cartilage degradation. Monitoring Lipid-ROS levels can aid in assessing OA progression and provide a basis for dynamically adjusting treatment strategies.

In the context of ferroptosis regulation, inhibiting Lipid-ROS generation is a crucial therapeutic approach for OA. Ferroptosis inhibitors like Fer-1 and Liproxstatin-1 (Lip-1) effectively reduce the accumulation of Lipid-ROS, thereby inhibiting chondrocyte ferroptosis and delaying OA progression (Skouta et al., 2014; Shah et al., 2017; Guo et al., 2022). While both Lip-1 and Fer-1 operate through similar mechanisms, Lip-1 possesses better pharmacokinetic properties, allowing it to inhibit ferroptosis effectively at lower doses (Wang et al., 2022c). However, these compounds have limitations in solubility and half-life, necessitating further optimization to enhance clinical applicability.

Melatonin has emerged as an important adjunct in combating OA due to its suppressing oxidative stress caused by iron overload (Yang et al., 2017). It inhibits ROS accumulation and protects stem cell osteogenic differentiation and proliferation by modulating signaling pathways like p53, ERK, and p38 (Yang et al., 2017). Other antioxidants also show potential in protecting cartilage by inhibiting Lipid-ROS. For instance, metformin reduces OA cartilage damage by inhibiting lipid peroxidation (Li et al., 2020); NAC prevents osteoblast apoptosis by inhibiting ROS production (Tian et al., 2016); resveratrol effectively reduces bone loss due to its potent antioxidant capabilities (Zhao et al., 2015); and tea polyphenols promote bone formation while reducing bone resorption through their antioxidant and anti-inflammatory properties (Shen et al., 2013). While these antioxidants hold great promise for OA treatment, further studies are required to validate their safety and efficacy before clinical application. Future therapeutic directions should focus on developing more targeted Lipid ROS inhibitors and utilizing advanced imaging techniques to monitor real-time dynamic changes in Lipid ROS levels.

### 4.3 Antioxidant defense-related biomarkers

#### 4.3.1 Nuclear factor E2-Related factor 2 (Nrf2)

Nrf2 is a key transcription factor that regulates the expression of antioxidant genes, directly influencing a cell’s antioxidant capacity. In OA, one of the core mechanisms of ferroptosis is the imbalance between lipid peroxidation and the antioxidant defense system. Nrf2 can effectively inhibit lipid peroxidation and mitigate cellular damage by regulating the expression of antioxidant enzymes such as GPX4. Therefore, activating Nrf2 offers a potential therapeutic strategy for intervening in ferroptosis in OA.

Studies have shown that Nrf2 activators, such as sulforaphane (SFN), can significantly reduce the generation of lipid peroxidation products by increasing GPX4 levels, thereby alleviating ferroptosis-induced damage to chondrocytes (Calabrese and Kozumbo, 2021; Mahn and Castillo, 2021). As a potent antioxidant, Pyrroloquinoline quinone (PQQ) may also reduce lipid peroxidation, though its direct impact on GPX4 regulation remains further elucidated (Li J et al., 2023). Additionally, ergosterol (ER) has been found to enhance Nrf2 activity, inhibiting molecules related to ferroptosis, such as MMP-9 and MMP-13, which strengthens the cell’s resistance to oxidative stress and protects chondrocytes from damage (Cai et al., 2021). Preclinical studies have validated the safety of Nrf2 activators, laying the groundwork for their clinical application in OA.

#### 4.3.2 Superoxide dismutase (SOD) and catalase (CAT)

SOD and CAT are essential enzymes in the antioxidant defense system. SOD converts superoxide anions (O₂⁻) into hydrogen peroxide (H₂O₂), which is then decomposed by CAT into water and oxygen, thereby alleviating oxidative stress. In osteoarthritis (OA), the inhibition of the Nrf2 pathway leads to decreased activity levels of SOD and CAT, impairing the antioxidant defense system’s ability to eliminate reactive ROS and increasing the risk of ferroptosis effectively. Since SOD and CAT neutralize excess ROS, their reduced activity directly reflects weakened antioxidant capacity and exacerbated ferroptosis.

Therefore, monitoring the activity levels of SOD and CAT can serve as supplementary indicators for assessing the antioxidant defense status and ferroptosis risk in OA patients. The significant roles of SOD and CAT in antioxidant defense and ferroptosis suppression also highlight the therapeutic potential of combining antioxidants with ferroptosis inhibitors. Employing a multi-target intervention strategy that includes anti-inflammatory drugs, antioxidants, and ferroptosis inhibitors can more effectively control oxidative stress and the ferroptosis process.

In terms of personalized treatment, adjusting therapeutic strategies based on the activity levels of SOD and CAT can optimize the suppression of ferroptosis according to the patient’s antioxidant capacity. Future research should focus on exploring the regulatory mechanisms of SOD and CAT, particularly on how to restore antioxidant defense capabilities when the Nrf2 pathway is inhibited in order to halt the progression of OA.

#### 4.3.3 Hypoxia-inducible factor - 2α (HIF-2α)

HIF-2α plays a significant role in the progression of OA, particularly in regulating the sensitivity of chondrocytes to ferroptosis. Studies indicate that HIF-2α, upon stimulation by inflammatory factors such as IL-1β, leads to a decrease in GSH levels, thereby increasing chondrocytes’ sensitivity to lipid peroxidation and ferroptosis. This process weakens the cellular antioxidant defense, accelerating chondrocyte death and the progression of OA pathology. Research has also shown that supplementing D-mannose can reverse this change, restoring GSH levels and enhancing the expression of GPX4 and SLC7A11, which reduces the occurrence of ferroptosis (Zhou et al., 2021).

The weakening of cellular antioxidant defenses caused by reduced GSH levels accelerates chondrocyte death and drives the progression of OA pathology. Future therapeutic strategies targeting HIF-2α-related pathways to modulate chondrocyte ferroptosis responses may effectively inhibit OA progression. Additionally, the potential of combining HIF-2α with other ferroptosis inhibitors warrants further exploration. By regulating oxidative stress responses within cartilage, combination therapies could more effectively address the sensitivity issues associated with HIF-2α-mediated ferroptosis and suppress OA deterioration. Clinical research should further validate the potential application of HIF-2α in personalized treatment, alongside ferroptosis markers like GPX4, to precisely regulate the survival environment of chondrocytes, advancing and refining OA treatment.

#### 4.3.4 Mitochondrial ferritin (FtMt)

FtMt, localized in mitochondria, is essential for maintaining iron homeostasis by reducing free iron ions and mitigating oxidative stress, thereby inhibiting ferroptosis (Wang et al., 2022b). FtMt deficiency has been shown to cause mitochondrial iron overload and oxidative stress, triggering ferroptosis through enhanced mitophagy (Wang et al., 2022b). In OA, these processes may contribute to cellular damage and disease progression. Ferroptosis inhibitors, such as Fer-1, have demonstrated the potential to alleviate oxidative stress, restore mitochondrial integrity, and reduce lipid peroxidation damage (Chen J et al., 2024). Additionally, significant rescue of IL-1β-induced loss of mitochondrial membrane potential is achieved by inhibiting hypoxia-inducible factor 1α (HIF-1α) or TfR1, indicating that protecting mitochondrial function plays a crucial role in preventing chondrocyte ferroptosis and degeneration (Chen B. Y. et al., 2024). Research has shown that Catalpol, an active component of traditional medicine, can promote mitochondrial biogenesis by inducing the expression of key regulators like PGC-1α, NRF1, and TFAM (Chen et al., 2022). It also enhances the expression of mitochondrial and nuclear DNA, increasing mitochondrial respiration rates and ATP production (Chen et al., 2022). These findings suggest that Catalpol may alleviate oxidative stress and ferroptosis by enhancing mitochondrial function, which could positively influence OA progression. Recent studies indicate that astragalus polysaccharides (APS) can significantly mitigate the damage caused by iron overload to the function of bone marrow mesenchymal stem cells by suppressing ROS levels within mitochondria (F. Yang et al., 2016). These findings further support the therapeutic potential of improving chondrocyte health in OA through mitochondrial function regulation.

In addition to conventional antioxidant and iron regulation strategies, botulinum toxin type A (BoNT/A) has emerged as a promising therapeutic approach for alleviating osteoarthritis (OA) by inhibiting chondrocyte ferroptosis. The mechanisms of BoNT/A include reducing reactive oxygen species (ROS) and iron ion accumulation, restoring mitochondrial function, and activating the SLC7A11/GPX4 anti-ferroptotic pathway (Zeng et al., 2024). Furthermore, the successful application of BoNT/A in experimental models offers valuable insights into its potential clinical translation in OA treatment. Future studies exploring the detailed mechanisms and long-term effects of BoNT/A may pave the way for precision OA therapy targeting ferroptosis regulation.

In summary, the ferroptosis-related biological molecules discussed in this section are presented in Table 2, while the small molecules and drugs are listed in Table 3.

**TABLE 3:** Ferroptosis-related small molecules and drugs in osteoarthritis.

Small molecules or drugs	Intervention target	Function	Reference
Malondialdehyde (MDA)	Cell membrane lipids	Toxic product that damages cell membrane integrity and increases permeability	Grigolo et al. (2003), Shah et al. (2005), Gladkova (2022)
4-Hydroxy-trans-2-nonenal (4-HNE)	Cell membrane lipids	Toxic product that damages cell membrane integrity and increases permeability	Grigolo et al. (2003), Shah et al. (2005), Gladkova (2022)
CoQ10	Mitochondrial respiratory chain, liposomal membranes	Inhibits lipid peroxidation	Frei et al. (1990)
BH4	liposomal membranes	Inhibits lipid peroxidation	Soula et al. (2020), Fanet et al. (2021)
Ferric Ammonium Citrate (FAC)	Cellular iron metabolism and cartilage homeostasis	Enhances iron-induced cell death and cartilage degradation	Yao et al. (2021)
PGE2	FLS	Enhances inflammation response	Tsai et al. (2017)
Ferristatin II	TfR1	Promotes TfR1 degradation and interferes with Tf-mediated iron delivery, intervening in OA pathology	(Cheng et al., 2022)
Biochanin A (BCA)	TfR1, FPN	Inhibits TfR1, promotes FPN expression, reduces intracellular iron concentration, alleviates mitochondrial damage and apoptosis in chondrocytes caused by iron overload	(He Q et al., 2023)
Deferoxamine (DFO)	Iron ions, MMP-1, MMP-13, IL-1β, TNF-α, NCOA4	Inhibits Type II collagen degradation, downregulates inflammatory cytokines, and restores iron homeostasis by chelating excess iron	Tchetina et al. (2016)
Deferiprone (DFP)	Iron ions	Chelates iron to reduce iron levels and prevent ferroptosis	Rodrigues de Morais and Gambero (2019)
Deferasirox (DFS)	Iron ions	Chelates iron to reduce iron levels and prevent ferroptosis	Rodrigues de Morais and Gambero (2019)
White Cardamonin (CAR)	SIRT1, p38MAPK, NLRP3 inflammasome, Type II collagen, MMP	Protects cartilage by reversing iron overload-induced chondrocyte damage and inhibiting apoptosis, reduces cartilage degradation and inflammation	(Li S et al., 2023)
YL-939	PHB2 (Prohibitin 2)	Regulates ferritin expression and phagocytosis to reduce intracellular free iron levels	Yang et al. (2022a)
Astaxanthin	p53 expression	Downregulates p53 expression and reduces ferroptosis-related protein expression	Wang et al. (2022a)
Tanshinone IIA (Tan IIA)	Oxidative stress (iron, ROS, MDA levels)	Reduces iron, ROS, and MDA levels, increases GSH levels, and improves oxidative stress status	(Xu J et al., 2023)
Naringin (NAR)	NRF2 expression	Reduces MDA and ROS accumulation, upregulates NRF2, mitigates oxidative stress	Pan et al. (2022)
Icariin (ICA)	GPX4	Upregulates GPX4 expression, reducing MDA and iron levels, activating System Xc^−^/GPX4 axis, inhibiting ferroptosis	(Wang et al., 2016; Xiao et al., 2024)
Thiazolidinedione (TZD)	ACSL4	Selective inhibition of ACSL4 activity, significantly inhibiting lipid peroxidation	Doll et al. (2017)
Ferrostatin-1 (Fer-1)	GPX4, Mitochondrial structure	Inhibits ferroptosis, upregulating GPX4 and SLC7A11, downregulating ACSL4 in mild OA\Restores mitochondrial structure	(Xu Y et al., 2023)
Liproxstatin-1 (Lip-1)	GPX4	Antioxidant enhancing GPX4 protective effects, reducing lipid peroxidation	(Skouta et al., 2014; Shah et al., 2017; Guo et al., 2022)
Vitamin E	GPX4	Antioxidant enhancing GPX4 protective effects, reducing lipid peroxidation	(Kagan et al., 2017; Homma et al., 2019)
Edaravone	GPX4	Antioxidant enhancing GPX4 protective effects, reducing lipid peroxidation	(Kagan et al., 2017; Homma et al., 2019)
N-acetylcysteine (NAC)	GPX4	Antioxidant enhancing GPX4 protective effects, delaying disease progression	Tian et al. (2016)
Melatonin	p53/ERK/p38	Blocks ROS accumulation and p53/ERK/p38 activation to protect against iron overload-induced osteogenic differentiation dysfunction and senescence	Yang et al. (2017)
Metformin	AMPK	Increased the expression of phosphorylated and total AMPK in articular cartilage tissue	Li et al. (2020)
Resveratrol	FOXO1, OPG/RANKL	Reverses the reduction of Runx2, OCN, and type I collagen caused by excess iron. Upregulates the level of FOXO1. Maintains the antioxidant/prooxidant equilibrium. Reduces the ratio of OPG/RANKL to inhibit osteoclastogenesis	(Zhao et al., 2015)
Tea polyphenols	Runx2, Wnt\caspase-3, RANKL	Promote bone formation by upregulating factors like Runx2 and Wnt, and inhibit bone resorption by reducing caspase-3 and RANKL	Shen et al. (2013)
Pyrroloquinoline quinone (PQQ)	Nrf2	Increases GPX4 levels, reduces lipid peroxidation, and mitigates ferroptosis damage to chondrocytes	(Li J et al., 2023)
Sulforaphane	Nrf2/ARE	Increases GPX4 levels, reduces lipid peroxidation, and mitigates ferroptosis damage to chondrocytes	(Calabrese and Kozumbo, 2021; Mahn and Castillo, 2021)
Ergosterol (ER)	Nrf2	Upregulates Nrf2 activity inhibits ferroptosis-related molecules such as MMP-9 and MMP-13, enhances oxidative stress resistance, and protects chondrocytes	Cai et al. (2021)
D-Mannose	HIF-2β	Restores GSH levels, increases GPX4 and SLC7A11 expression, reducing ferroptosis in chondrocytes	Zhou et al. (2021)
Catalpol	Mitochondrial biogenesis (PGC-1α, NRF1, TFAM)	Promotes mitochondrial DNA and nuclear DNA expression, enhances mitochondrial respiration rate and ATP production	Chen et al. (2022)
Astragalus Polysaccharides (APS)	Mitochondrial ROS levels	Mitigates iron overload-induced damage in bone marrow mesenchymal stem cells	Yang and Stockwell (2016)

## 5 Discussion and perspectives

Osteoarthritis (OA) is a common degenerative condition, marked by gradual progression and associated with high rates of disability, resulting from complex etiological factors that limit effective treatment options. Abnormal iron metabolism significantly impacts bone and joint health, highlighting the importance of maintaining iron homeostasis for the integrity of joint structures. Ferroptosis, a novel form of cell death associated with iron imbalance, reactive oxygen species (ROS) accumulation, and mitochondrial membrane damage, is closely linked to the progression of OA. However, the mechanisms underlying ferroptosis remain complex and not fully elucidated. Extensive research has identified key molecules and pathways in ferroptosis, such as GPX4, iron metabolism, and lipid peroxidation. However, modulating these mechanisms in the complex OA microenvironment across various cell types and tissues remains challenging.

Currently, ferroptosis primarily influences the pathological progression of OA indirectly through iron overload. Future research should focus more on its direct mechanisms. Past studies have predominantly concentrated on cartilage tissue, yet cartilage lacks nerve distribution; thus, pain is more likely associated with vascular and neural invasion in subchondral bone and synovial inflammation. Future investigations could explore novel therapeutic avenues from the synovium and subchondral bone perspectives.

Current ferroptosis inhibitors like GPX4 agonists and iron chelators show promise in experiments and clinical trials. However, targeting specific cells or tissues remains a challenge. Non-specific treatments may lead to side effects or impair other physiological functions, limiting clinical applicability. Therefore, precisely regulating ferroptosis targets without compromising normal cell functions is a critical challenge for future therapies. Employing multi-omics technologies to identify ferroptosis-related molecular biomarkers, coupled with large-scale clinical trials, will aid in validating the efficacy of ferroptosis inhibitors and promote their clinical application.

Innovative therapeutic strategies targeting ferroptosis are essential. Developing novel ferroptosis inhibitors and intervention methods, such as small molecule drugs, gene therapy, and cell therapy, may offer more effective treatment options for OA in the context of ferroptosis. Embracing the concept of precision medicine through personalized treatment approaches will also be a significant future direction to enhance efficacy and safety. Comprehensive treatment strategies that integrate various therapeutic modalities may yield improved outcomes. Future studies should investigate the combined use of ferroptosis inhibitors with other therapies, such as anti-inflammatory drugs and immunomodulators, to enhance treatment effectiveness.

This review elucidates the molecular mechanisms of ferroptosis in OA and its critical role in disease progression, exploring the impacts of iron metabolism dysregulation, lipid peroxidation, and the collapse of antioxidant defense systems on cartilage degeneration, synovial inflammation, and subchondral bone remodeling. It highlights the potential of key molecules like GPX4, TfR1, and NCOA4 as diagnostic and therapeutic targets. However, this review has limitations, notably its inability to comprehensively cover the specific molecular mechanisms of ferroptosis across different OA subtypes, and it provides limited discussion on the interactions between ferroptosis and other forms of cell death. Future research should deepen the understanding of ferroptosis in various OA subtypes, identify additional key regulatory factors and signaling pathways, and explore the interactions between ferroptosis and other cell death modalities, such as apoptosis and necrosis. Overall, the study of ferroptosis in OA presents vast potential. Through ongoing research and exploration, we anticipate achieving precise regulation of ferroptosis, developing more effective treatment methods, and ultimately providing new solutions for managing OA, thereby improving patients’ quality of life and health outcomes.
